# Pilocarpine in Cutaneous Diseases

**Published:** 1881-03

**Authors:** 


Pilocarpine in Cutaneous Diseases.—J. Pret, (*Centralblatt fur Chirurgie,* No. 2, 1881,) has been experimenting with pilocarpine in chronic dermatoses on a theory of securing a permanent influence on the secretions of the skin. The dose ordinarily given was one-sixth of a grain once or twice daily, the patient being allowed to go about. In case more extended perspiration was desired the remedy was administered in larger doses, the patient being confined to bed.

Prurigo improved markedly under the use of the remedy in a short time, the ichorous discharge diminishing and the disease being removed in four to eight weeks. The disease at times had a very mild relapse. In psorias the remedy exerted no influence, while moist, or acute dry eczema were injuriously influenced by it. Chronic eczema were much benefitted by it. Pruritus vulva and senilis were treated successfully. A case of chronic urticara in which arsenic and similar remedies had been uselessly tried for several weeks was relieved in eight days. In aleopecia areata, four to five months elapsed before the hair appeared. In aleopecia pityroides a decidedly favorable result was obtained. These cases certainly seem to show that pilocarpine is of value in the dermatoses; however, many of the cases given have elements of error about them that render further experiment necessary.—*Chicago Medical Review.*

